# Natural history of *Acinetobacter baumannii* infection in mice

**DOI:** 10.1371/journal.pone.0219824

**Published:** 2019-07-18

**Authors:** Brian M. Luna, Jun Yan, Zeferino Reyna, Eugene Moon, Travis B. Nielsen, Hernan Reza, Peggy Lu, Robert Bonomo, Arnold Louie, George Drusano, Jürgen Bulitta, Rosemary She, Brad Spellberg

**Affiliations:** 1 Department of Medicine, Keck School of Medicine at the University of Southern California (USC), Los Angeles, California, United States of America; 2 Department of Molecular Microbiology and Immunology, Keck School of Medicine at the University of Southern California (USC), Los Angeles, California, United States of America; 3 Department of Pathology, Keck School of Medicine at the University of Southern California (USC), Los Angeles, California, United States of America; 4 Departments of Medicine, Pharmacology, and Molecular Biology and Microbiology, Louis Stokes Cleveland Department of Veterans Affairs Medical Center, Case Western Reserve University, Cleveland, Ohio, United States of America; 5 Center for Pharmacometrics & Systems Pharmacology, Department of Pharmaceutics, College of Pharmacy, University of Florida, Orlando, Florida, United States of America; 6 Institute for Therapeutic Innovation, College of Medicine, University of Florida, Orlando, Florida, United States of America; Tulane University School of Medicine, UNITED STATES

## Abstract

In 2017, the WHO identified *Acinetobacter baumannii* as the top priority for the development of new antibiotics. Despite the need for new antibiotics, there remains a lack of well validated preclinical tools for *A*. *baumannii*. Here, we characterize and validate a mouse model for *A*. *baumannii* translational research. Antibiotic sensitivity for meropenem, amikacin, and polymyxin b was determined by the broth microdilution MIC assay. LD_100_ inoculums, in both blood and lung infection models, were determined in male and female C3HeB/FeJ mice that were challenged with various *A*. *baumannii* clinical isolates. Blood (blood infection model) or blood and lung tissue (lung infection model) were collected from infected mice at 2 and 18 hours and the bacterial burden was determined by quantitative culture. Blood chemistry was analyzed using the iStat system. Cytokines (IL-1ß, TNF, IL-6, and IL-10) were measured in the blood and lung homogenate by ELISA assay. Lung sections (H&E stains) were scored by a pathologist. In the blood infection model, the cytokines and physiological data indicate that mice become moribund due to sepsis (low blood pH, falling bicarbonate, and a rising base deficit), whereas mice become moribund due to respiratory failure (low blood pH, rising bicarbonate, and a falling base deficit) in the oral aspiration pneumonia model. We also characterized the timing of changes in various clinical and biomarker endpoints, which can serve as a basis for future interventional studies. Susceptibility was generally similar across gender and infection route. However, we did observe that female mice were approximately 2-fold more sensitive to LAC-4 Col^R^ in the blood infection model. We also observed that female mice were more than 10-fold more resistant to VA-AB41 in the oral aspiration pneumonia model. These results establish parameters to follow in order to assess efficacy of novel preventative and therapeutic approaches for these infections.

## Introduction

The World Health Organization (WHO) recently released a list of priority pathogens for which new antibiotics are most needed and carbapenem-resistant *Acinetobacter baumannii* was listed as a “Priority 1: Critical Pathogen.”[1,2] The current pipeline of novel therapeutics is inadequate, and particularly so for *A*. *baumannii*.[1–3] One potential obstacle that is continuing to hinder drug development efforts are the lack of validated animal models for the preclinical study of *A*. *baumannii* infections.

Numerous animal studies have previously been published related to *A*. *baumannii*, however many studies use infection strategies that raise concerns regarding their clinical relevance.[4–7] For example, many studies use neutropenic mice to increase their susceptibility to infection,[8–10] despite the fact that neutropenia is an uncommon risk factor for *A*. *baumannii* infection in patients.[2,11–14] Other studies have used routes of infection (e.g., intraperitoneal) that do not recapitulate clinical disease, and many studies have used *A*. *baumannii* strains that are laboratory adapted and/or unable to cause lethal infection at reasonable infectious inocula.[2]

Here we extensively characterize the natural history of disease caused by *A*. *baumannii* infection, for both bacteremia and aspiration pneumonia, in normal, immune competent mice. We describe *A*. *baumannii* clinical isolates that are virulent in blood and lung infection model in mice, with different antimicrobial sensitivity profiles including resistance to amikacin, colistin, and/or meropenem. These studies of defined bacteria isolates/mouse model pairings will enable preclinical studies to identify promising new therapeutic candidates for clinical development.

## Materials and methods

### Ethics statement

All animal work was conducted following approval by the Institutional Animal Care and Use Committee (IACUC protocol 20810) at the University of Southern California, in compliance with the recommendations in the Guide for the Care and Use of Laboratory Animals of the National Institutes of Health. Infected mice develop weight loss, ruffled fur, poor appetite, decreased ambulation, huddling behavior, and low body temperature. Mice that display huddling behavior and are poorly mobile will be weighed 1x daily. Weight loss of greater than 15% body weight will trigger euthanasia. Mice were monitored at least twice daily for seven days. Soft bedding and other enrichment devices were provided as recommended by the vet staff. Nutritional supplements, such as the hydrogel packs were provided as needed.

### Bacteria culture

We identified virulent clinical isolates of *A*. *baumannii* with varying antimicrobial susceptibilities to key antibiotics (Table 1). Overnight cultures of *A*. *baumannii* were grown in Tryptic Soy Broth (TSB) at 37°C. The overnight culture was diluted 1:100 and then subcultured in MHII at 37°C / 200 rpm until the culture reached an OD_600_ of 0.5. The log-phase culture was diluted to the target inoculum prior to infection. The inoculum was determined by plating serial dilutions on TSA plates.

**Table 1:** Summary of strains used in this study.

		MIC (μg/ml)		
*A*. *baumannii* Strain	Origin	Amikacin	Meropenem	Polymyxin B
**HUMC1**	**Blood and lung [Bruhn ‘14 JID]**	128	128	0.25
**VA-AB41**	**Skin, lung [Perez ‘10 ]**	8	64	0.50
**LAC-4**	**Blood [Valentine ‘08 J Clin Micro]**	64	1	0.25
**LAC-4 Col**^**R**^	**Lab passage for this study**	128	4	64
**ATCC 17978**	**Remote clinical CSF, lab attenuated [Piechud ‘51 Ann Inst Pasteur]**	8	0.25	0.5
**C-14**	**Wound [Arroyo ‘11 AAC]**	2	1	8
**C-8**	**Blood [Arroyo ‘11 AAC]**	8	8	16

### MIC protocol

MHII media was used for the minimum inhibitory concentration (MIC) assays, per Clinical Laboratory Standards Institute (CLSI) guidance. All of the MICs were performed in sterile 96-well round bottom microwell plates. Briefly, 100 μl of MHII media was added to the wells in columns 2–10. Next, 200 μl of a 2X antibiotic working solution was added to the wells in column 1. Two-fold serial dilutions of the antibiotic were performed through column 10. Next, 100 μl of a 1×10^6^ CFU/mL working solution of bacteria was added to each of the wells in columns 1–11. Column 11 served as a bacteria-only positive control and column 12 was a media-only negative control. The inoculum concentration was confirmed by plating serial dilutions on TSA plates.

### Antibiotic preparation

A fresh stock of amikacin, meropenem, or polymyxin b was prepared daily for the MIC assay. For *in vitro* testing, the stock solution was prepared by dissolving the drugs in molecular grade sterile water. The working solution of antibiotic was prepared 2X of the desired starting well final drug concentration.

### Mouse studies

Healthy male and female, immune normal C3HeB/FeJ (Jackson Laboratory, stock no. 000658) mice were used, as we and others have previously found that such mice are more susceptible to infection caused by *A*. *baumannii* than other commonly used inbred strains.[2]

#### Intravenous (IV) infection

Bacterial inocula, made from fresh or frozen bacteria, was prepared as described in previous work.[15] Frozen stocks of HUMC1, VA-AB41, and LAC-4 Col^R^ were thawed and diluted in PBS to adjust the bacterial density as needed for infection. 8-week old male and 10-week old female C3HeB/FeJ mice were infected via tail vein injection and the inoculum bacterial density was confirmed by plating serial dilutions on TSA plates and incubating overnight at 37°C.

#### Oral aspiration (OA) pneumonia infection

Our pneumonia model was conducted as previously published.[16] Single colonies of *A*. *baumannii* grown on TSA were used to inoculated TSB and bacteria were cultured overnight at 37°C / 200 rpm. The following day, the bacteria was subcultured by diluting the overnight 1:100 in fresh TSB and cultured for 3hrs at 37°C / 200rpm. The subculture was washed with PBS three times and adjust to optical density (OD_600_) equal to 0.5. The inoculum was concentrated to 2x10^9^ CFUs/ml and 8-week old male and 10-week old female C3HeB/FeJ mouse is infected with 50 ul of inoculum via oral aspiration. The inoculum CFUs was confirmed by plating on TSA plates and incubating overnight at 37°C.

#### Biomarkers and clinical markers

A terminal cardiac puncture was performed after sedation with ketamine/xylazine to collect blood from mice at the time points indicated. For analysis of the blood chemistry, fresh blood was spotted on a the EC8+ cartridge (Abbott, 03P79-25) and analyzed using an iStat reader (Abbott). Internal body temperature was recorded using a rectal temperature probe. Activity scores were defined as follows: “4” = normal activity, “3” = diminished activity, “2” = no movement but walks when stimulated, and “1” = does not walk even when stimulated. The following cytokines were measured from the collected serum per manufacturer protocols: TNF (Thermo Fisher, 88-7324-76), IL-10 (Thermo Fisher, 88-7105-76), IL-1 beta (Thermo Fisher, 88-7013-76) and IL-6 (Thermo Fisher, 88-7064-76).

#### Histopathology

For OA infection model, whole lungs were collected at necropsy for both male and female mice at pre-infection and 2 hours and 18 hours post-infection. The lungs were fixed in 10% formalin and embedded in paraffin blocks, then cut sections underwent hematoxylin and eosin (H&E) staining. Histopathological analysis and interpretation was done by a trained pathologist.

#### Statistical analysis

Survival was compared by the nonparametric log-rank test. Bacterial density was compared with the Mann Whitney test for unpaired comparisons. All statistics were run using Graphpad Prism software. Differences were considered significant if the P value was < .05.

## Results

### Antibiotic sensitivity testing

Sensitivity testing ensured that we used clinical isolates of *A*. *baumannii* with broad representation of antibiotic-resistant and antibiotic-susceptible phenotypes, enabling a unique sensitivity profiles for downstream applications (Table 1). All together we compiled a set of strains that included resistance and sensitivity to amikacin (aminoglycoside), meropenem (carbapenem), and colistin (polymyxin). The LAC-4 Col^R^ strain was a spontaneous mutant that was derived from the LAC-4 parent strain by plating a high inoculum of bacteria on agar plates supplemented with 32 μg/mL colistin.

### Establishing LD100_s_

Prior to conducting *in vivo* virulence studies, we noted significant differences in body weight between male and female mice of the same age. To ensure mice of different genders had similar body mass for simultaneous infections, we tested various age groups and found that 8-week old males and 10-week old female mice had similar body weights (S1 Fig). Thus we used mice of these gender/ages for all experiments.

We initially sought to determine the 100% lethal inoculum (LD_100_) for the tail-vein bacteremia and oral aspiration pneumonia infection models. Mice were euthanized when they achieved moribund condition, which was defined as inability to take a purposeful step after a gentle pull of the mouse’s tail; mice who developed other conditions precluding normal ambulation (e.g., paralysis of one or more limbs, severe listing to one side of the body) were also considered to have achieved moribund condition and euthanized.

We found a >100-fold variance across strains in LD_100_s in the bacteremia model (Table 2). The LD_100_s for the pneumonia model were nearly 10-fold higher than the bacteremia model for the HUMC1 and VA-AB41 strains. However, the LD_100_ inoculums were similar between the bacteremia and pneumonia models for the LAC-4 and LAC-4 Col^R^ strains.

**Table 2:** LD100s of *A*. *baumannii* Isolates^1^.

	IV bacteremia model		OA pneumonia model	
	Sublethal CFU	LD_100_ CFU	Sublethal CFU	LD_100_ CFU
**HUMC1**	5.4x10^6^	2.0x10^7^	2.5x10^8^	4.7x10^8^
**LAC-4**	1.0x10^7^	2.5x10^7^	1.9x10^7^	2.8x10^7^
**LAC-4 Col**^**R**^	5.7x10^7^	9.7x10^7^	2.4x10^7^	7.6x10^7^
**VA-AB41**	1.0x10^7^	4.3x10^7^	3.2x10^8^	6.2x10^8^
**ATCC 17978**	5.0x10^8^	9.0x10^8^	N/A	N/A
**C-14**	5.0x10^8^	N/A	N/A	N/A
**C-8**	8.6x10^8^	9.6x10^8^	N/A	N/A

^1^The number shown reflect the highest sublethal inocula or lowest lethal inocula identified after multiple experiments were conducted for each strain. For each experiment N = 10 mice per group (5 males + 5 females). N/A indicates we could not achieve LD100s for the IV model, and did not attempt them for the OA model because the IV LD100s were so high/unachievable.

Of note, the colistin-resistant strains required a higher LD_100_ compared to colistin sensitive strains (Table 2). The change in LD_100_ was particularly striking when comparing the colistin-resistant LAC-4 Col^R^ strain (derived by serial passage in colistin *in vitro*) to its parent colistin-sensitive LAC-4 strain. The other colistin-resistant strains tested, C-8 and C-14 (both of which developed colistin-resistance during clinical therapy of patients), were also less virulent, requiring higher LD_100_s, in the bacteremia model. Given their diminished virulence, we did not pursue additional studies with C-8 and C-14.

**Fig 1:**
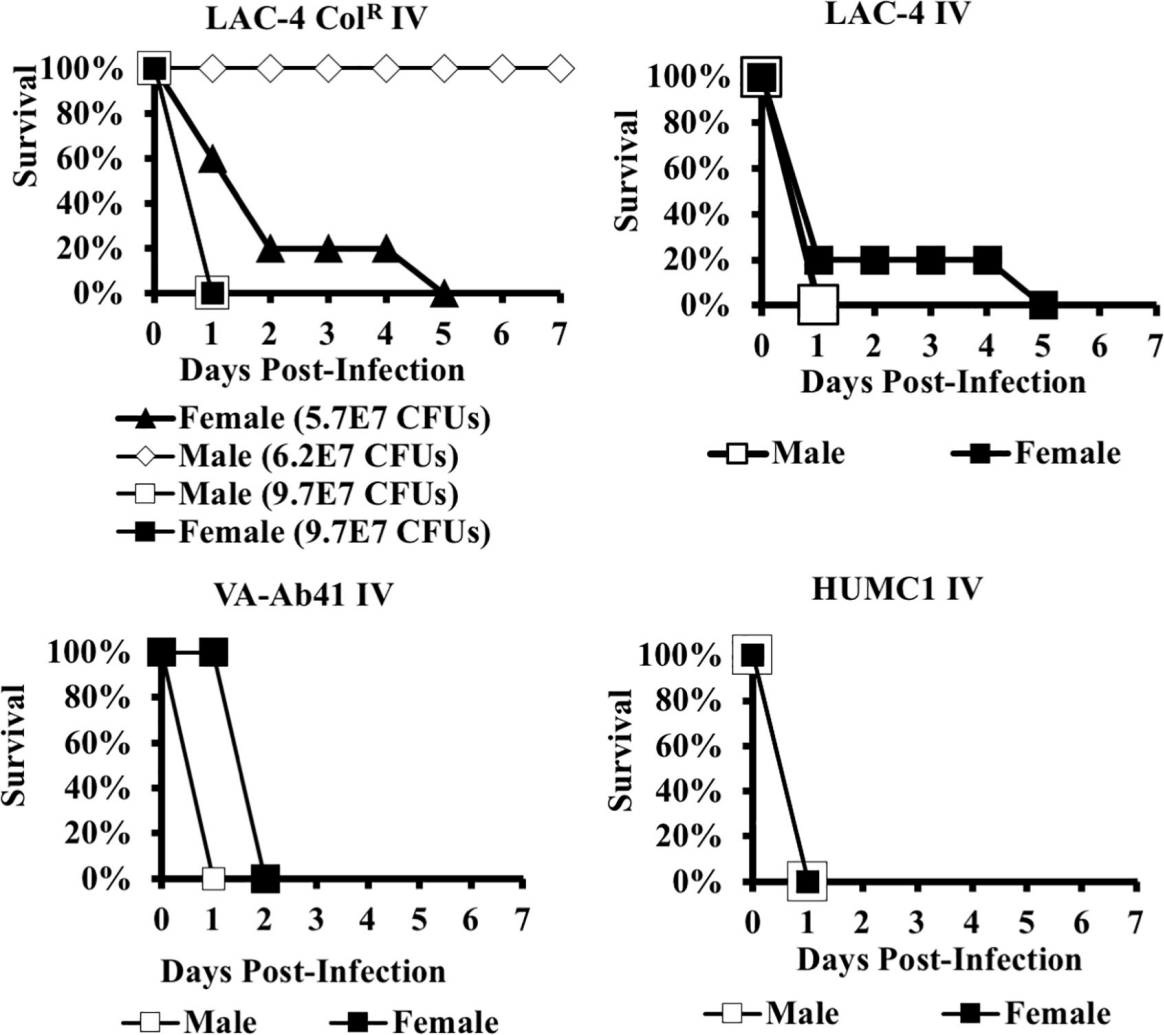
The effect of gender on mouse susceptibility, blood infection model. Mice were infected with the LD100 inoculum listed in Table 1 unless otherwise specified. Variation in host susceptibility by gender was not consistently observed across all strains or infection routes. In the blood infection model, a gender effect (female mice were more sensitive as compared to males) was only observed with mice infected with LAC-4 Col^R^.

**Fig 2:**
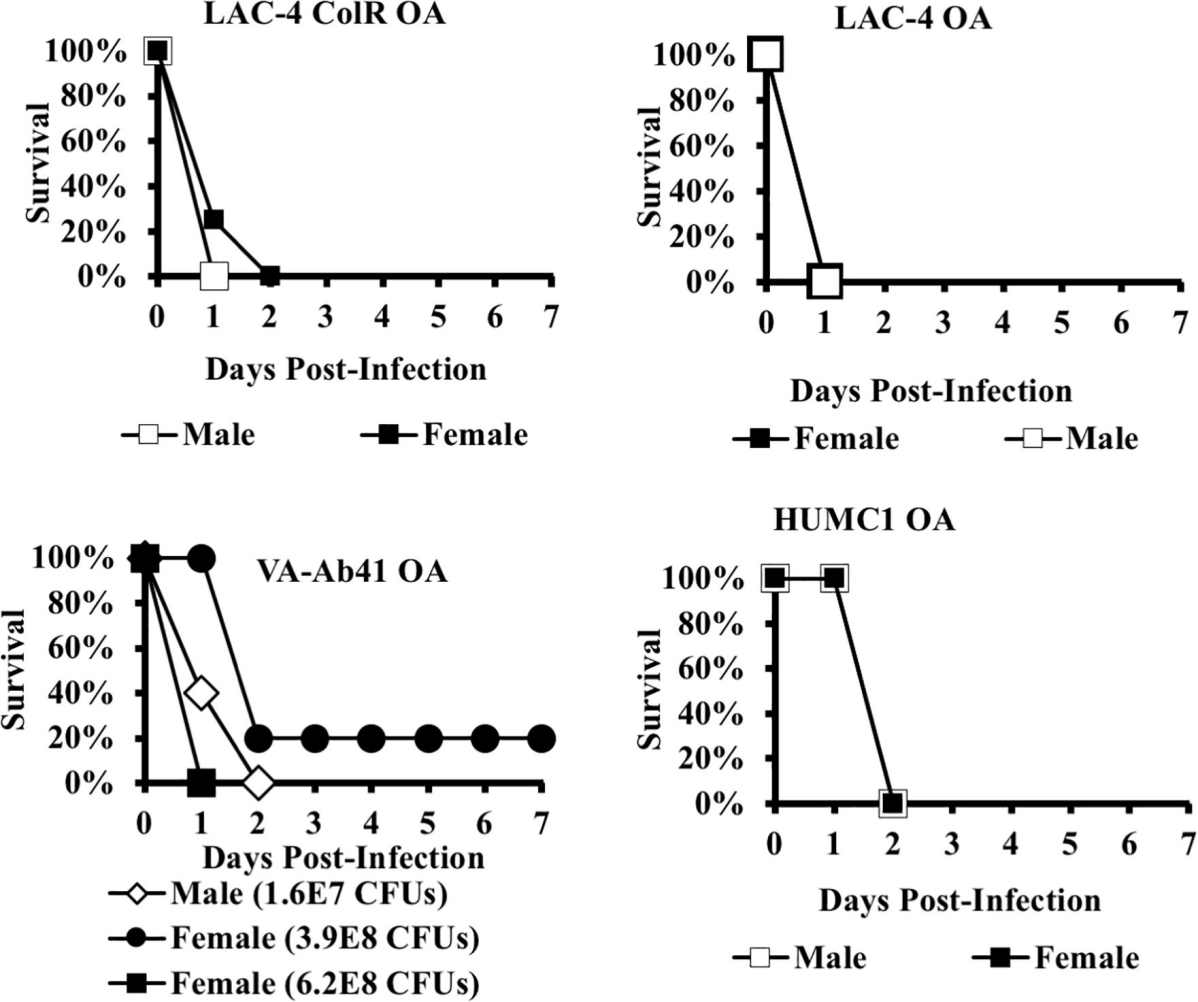
The effect of gender on mouse susceptibility, OA infection model. Mice were infected with the LD100 inoculum listed in Table 1 unless otherwise specified. Variation in host susceptibility by gender was not consistently observed across all strains or infection routes. In the OA infection model, a gender effect (males were more sensitive as compared to females) was only observed with mice infected with VA-AB41.

For most of the strains in either mouse model, we did not discern an effect of mouse gender on host outcome. However, we did discern a gender effect in the LD100s in mice infected with LAC-4 Col^R^ in the bacteremia model and in mice infected with VA-AB41 in the OA model (Figs 1 and 2). Specifically, the LD100 for female mice was approximately 2-fold lower than for male mice during IV infection with the LAC-4 Col^R^ strain. The gender effect was more pronounced (and in the opposite direction) for OA infection by VA-AB41, for which the LD100 for male mice more than 10-fold lower than for female mice.

### Microbiological and clinical endpoints

**Fig 3:**
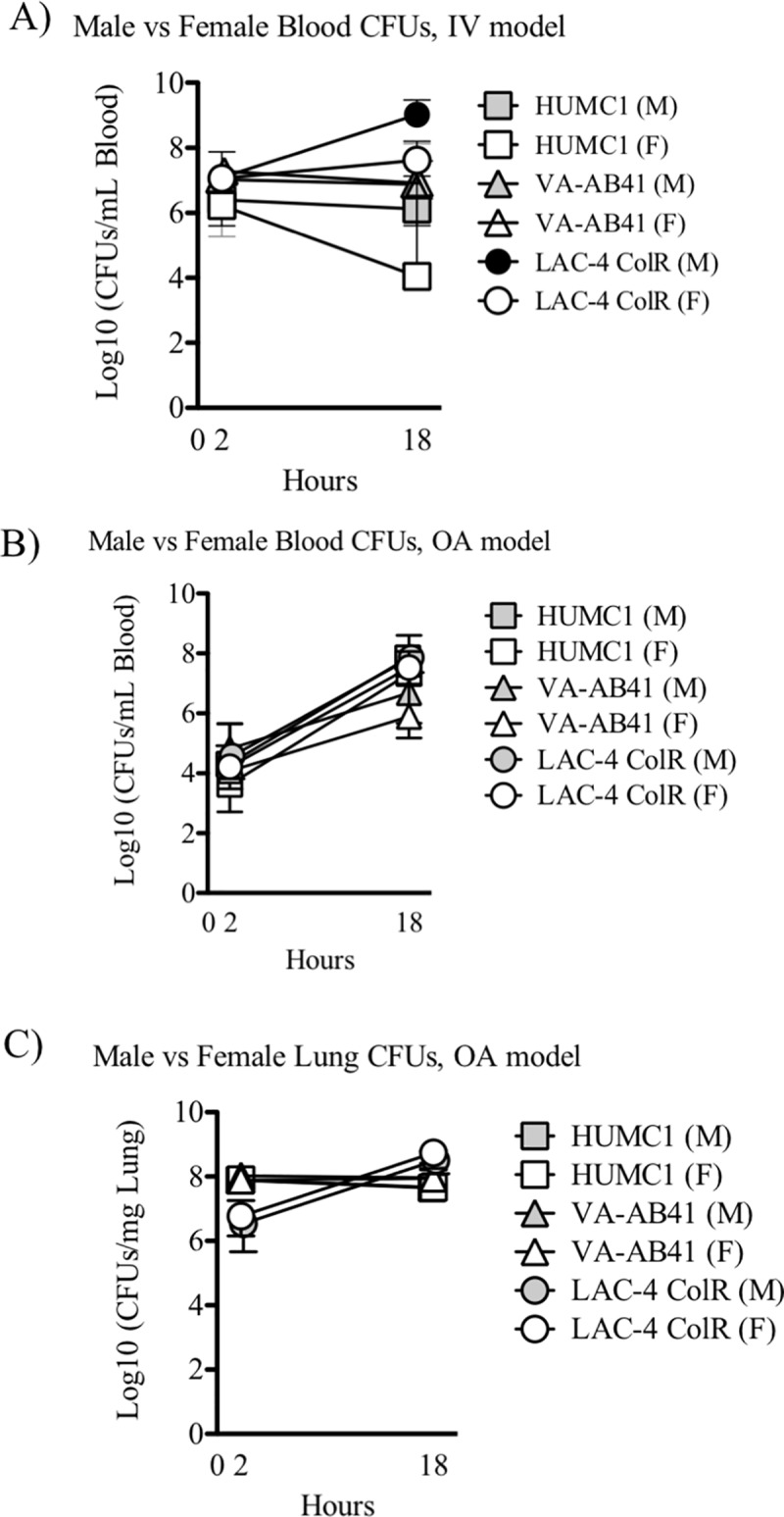
Blood and lung bacterial densities in male and female mice infected with *A*. *baumannii* strains. Male and female C3H/FeJ mice were challenged with *A*. *baumannii* strains HUMC1, VA-AB41, or LAC-4 Col^R^. **A)** Mice were infected IV with >LD100s for each strain: HUMC1 (2.0x10^7^ CFU), VA-AB41 (2.7x10^7^ CFU), LAC-4 Col^R^ (9.7x10^7^ CFU). Blood was collected at 2 and 18 hours and serial dilutions were plated to determine the bacterial burden. No significant difference was observed between gender. At 2 hours, a significant difference was observed between HUMC1 vs VA-AB41 and LAC-4 Col^R^ infected mice (P < .01). At 18 hours, a significant difference was observed between HUMC1 and LAC-4 Col^R^ infected mice (P < .01). **B)** Mice were challenged via the lung with >LD100s for each strain: HUMC1 (4.7x10^8^ CFU), VA-AB41 (6.2x10^8^ CFU), LAC-4 Col^R^ (7.6x10^7^ CFU). Blood (B) or lungs (C) were collected at 2 and 18 hours and quantitatively cultured. No significant difference was observed between gender for the blood CFUs. At 18 hours, VA-AB41 was significantly different compared to HUMC1 and LAC-4 Col^R^ (P<0.05). For the lung homogenate, a significant difference was observed at 2 hours for LAC-4 Col^R^ vs VA-AB41 and HUMC1 (P < 0.01). At 18 hrs, there was a significant difference between HUMC1 and LAC-4 Col^R^ infected mice (P<0.01).

The bacteremia infection model results in very rapid progression of illness, and mice are generally moribund within 24 hours. We therefore measured the bacterial burden at 2 and 18 hours post infection in mice infected with HUMC1, VA-AB41, and LAC-4 Col^R^ strains (Fig 3A). At 2 hours post-infection, blood bacterial density clustered between 10^6^ and 10^7^ CFUs/ml across all strains. Most strains maintained similar bacterial density between 2 and 18 hours; in contrast LAC-4 Col^R^ increased in bacterial density over that period of time, particularly in male mice, while HUMC1 decreased in bacterial density in female, but not male mice. As a result, despite the fact that all mice were lethally infected based on established LD100s, the bacterial blood densities varied by a remarkable 10^5^-fold at 18 hours after infection, indicating a substantial dissociation between bacterial density and lethality.

We have previously found that lethal aspiration pneumonia is accompanied by bacteremia.[17] In contrast to blood bacterial density in the bacteremia model, in the oral aspiration pneumonia model, blood bacterial burden uniformly increased across strains by 50- to 1000-fold between 2 and 18 hours post-infection, with no appreciable variance by mouse gender (Fig 3B). Lung CFUs in the model remained relatively stable across the strains, except for the LAC-4 Col^R^ strain, which increased approximately 100-fold in both male and female mice (Fig 3C).

**Fig 4:**
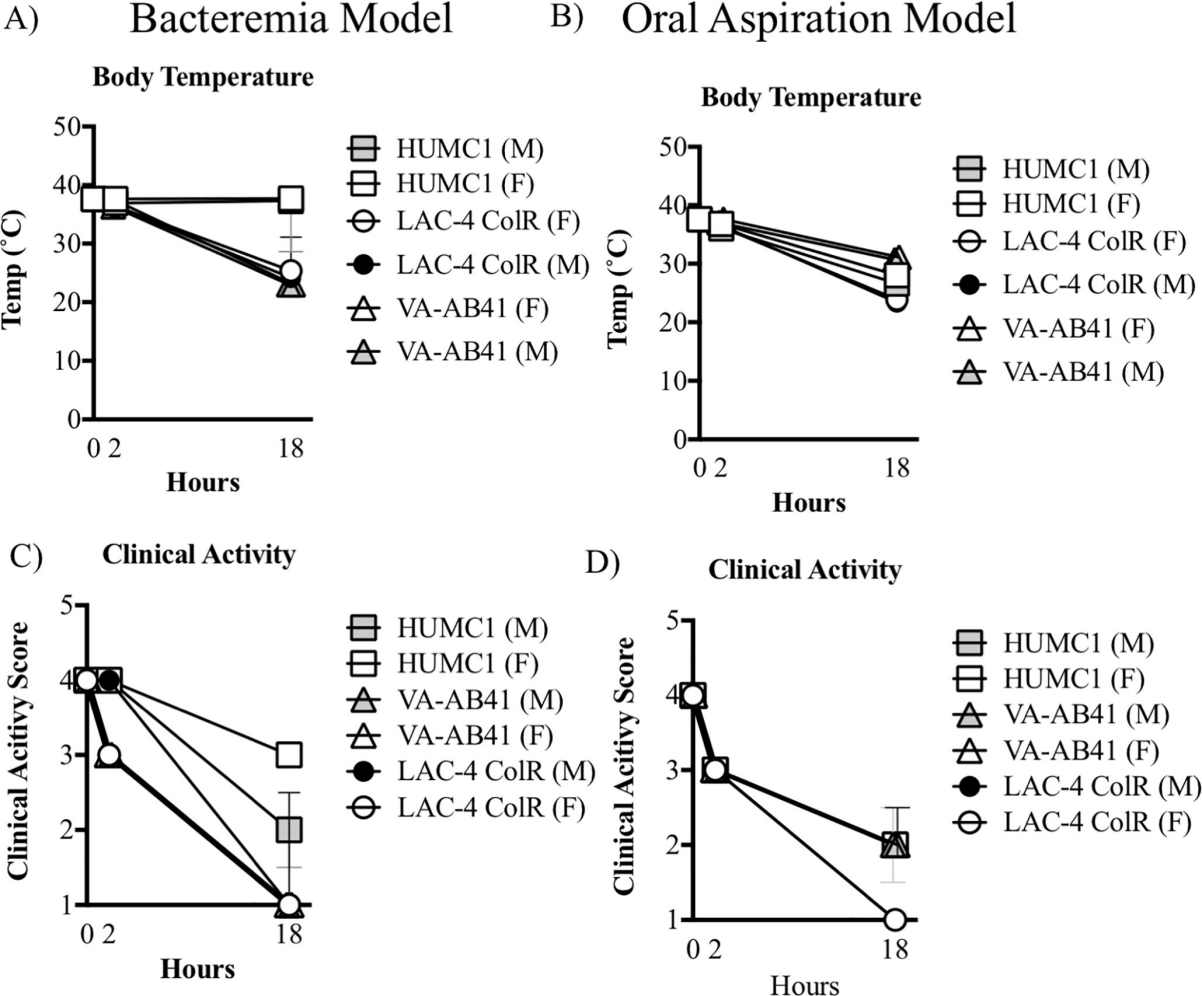
Temperature and clinical scores in infected mice. Male and female C3H/FeJ mice were challenged with with *A*. *baumannii* strains HUMC1, VA-AB41, or LAC-4 Col^R^. Mice were infected using the bacteremia and oral aspiration pneumonia models. Unless otherwise noted, no significant difference was observed between gender. **A)** At 2 hours, there was a significant difference between LAC-4 Col^R^ and. HUMC1 (P<0.05). At 18 hours, there was a significant difference between VA-AB41 and LAC-4 Col^R^ and HUMC1 (P<0.05). **B)** At both 2 and 18 hours, there was a significant difference between VA-AB41 vs. LAC-4 Col^R^ and HUMC1 (P<0.05). **C)** At 2 hours, there was a significant difference between gender for LAC-4 Col^R^ infected mice (P<0.05). (P<0.05). At 18 hours, a significant decrease was observed for all infecting strains (P<0.05). **D)** At both 2 and 18 hours, a significant decrease in activity was observed for all infecting strains (P<0.01).

Clinically, mice became progressively hypothermic and with worsening clinical activity scores over time in both models (Fig 4), despite the variance in bacterial densities across the mice. These results suggested that the inflammatory response was driving outcomes as much as bacterial density. Of note, female mice infected IV with LAC-4 Col^R^ had worse clinical activity scores than male mice at 2hours post-infection (Fig 4C), despite being uniformly lethally infected. Thus, the male mice exhibited delay in clinical deterioration to LAC-4 Col^R^ infection but by less than a day.

### Biomarkers

**Fig 5:**
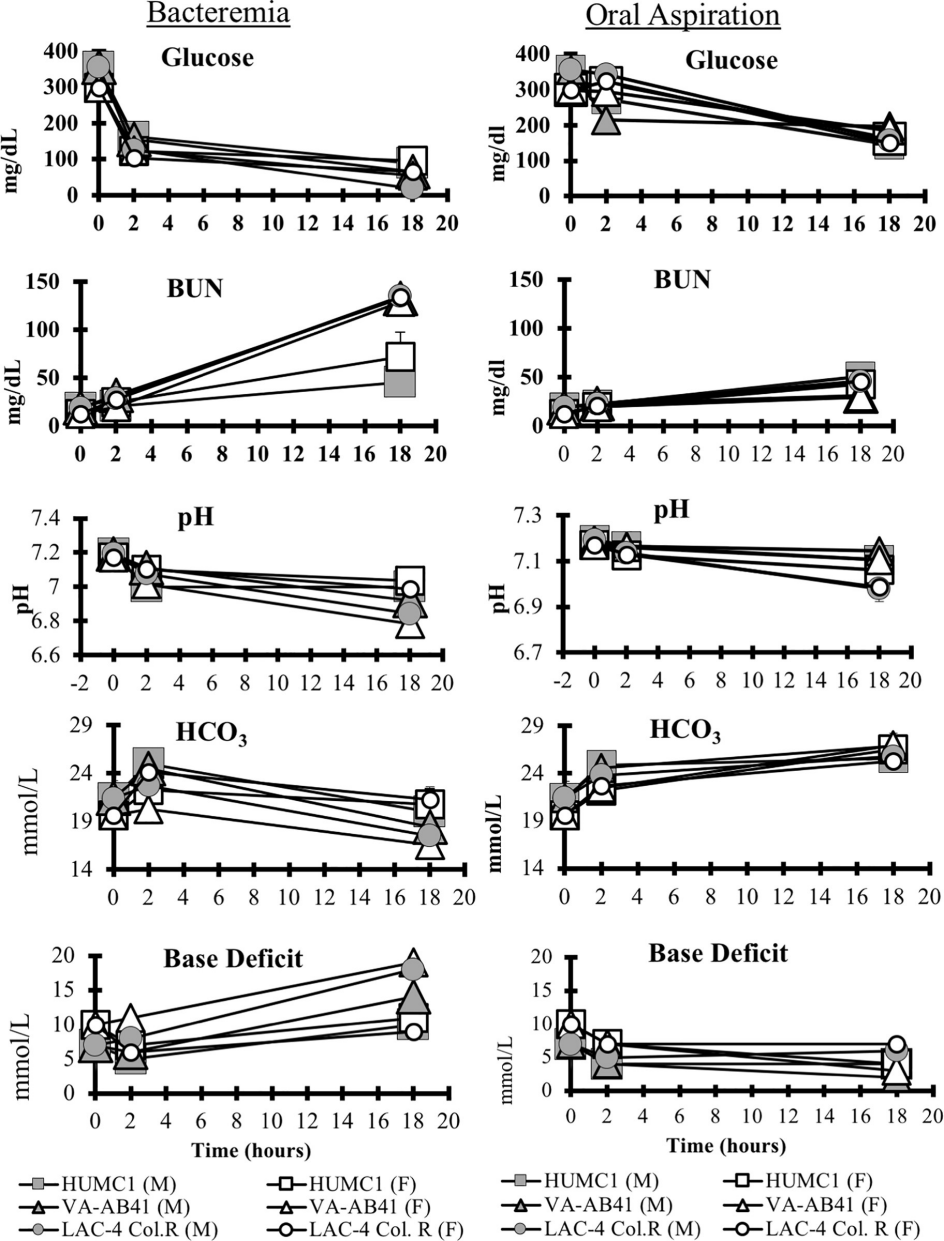
Sepsis biomarkers. Blood samples were collected from healthy and infected mice at 2 and 18 hours post-infection. BUN = blood urea nitrogen, a marker of renal function. Base excess = difference between measured and predicted serum bicarbonate. Falling base excess is consistent with a metabolic acidosis due to sepsis, while rising base deficit is consistent with respiratory failure, trapping CO2 in the blood, resulting in increased bicarbonate and carbonic acid levels. BUN was significantly increased at 18 hours for all infecting strains in the bacteremia model but only HUMC1 and VA-AB41 in the OA model (P<0.05). Bicarbonate was significantly increased in the OA infection morel for all infecting strains (P<0.05). Glucose was significantly decreased for all infecting strains in both the bacteremia and OA models at 18 hours post infection (P <0.05). The pH was significantly decreased in both infection models for only mice infected with LAC-4 Col^R^ (P < .001).

Across both models and all infecting strains, mice became progressively hypoglycemic, acidemic, and developed renal failure (rising blood urea nitrogen) (Fig 5), consistent with progressive sepsis. Interestingly, in the bacteremia model, falling blood pH was accompanied by falling bicarbonate and a rising base deficit (difference between measure bicarbonate levels and normal bicarbonate level), while in the pneumonia model, the opposite occurred, as the falling blood pH was accompanied by rising bicarbonate and falling base deficit.

**Fig 6:**
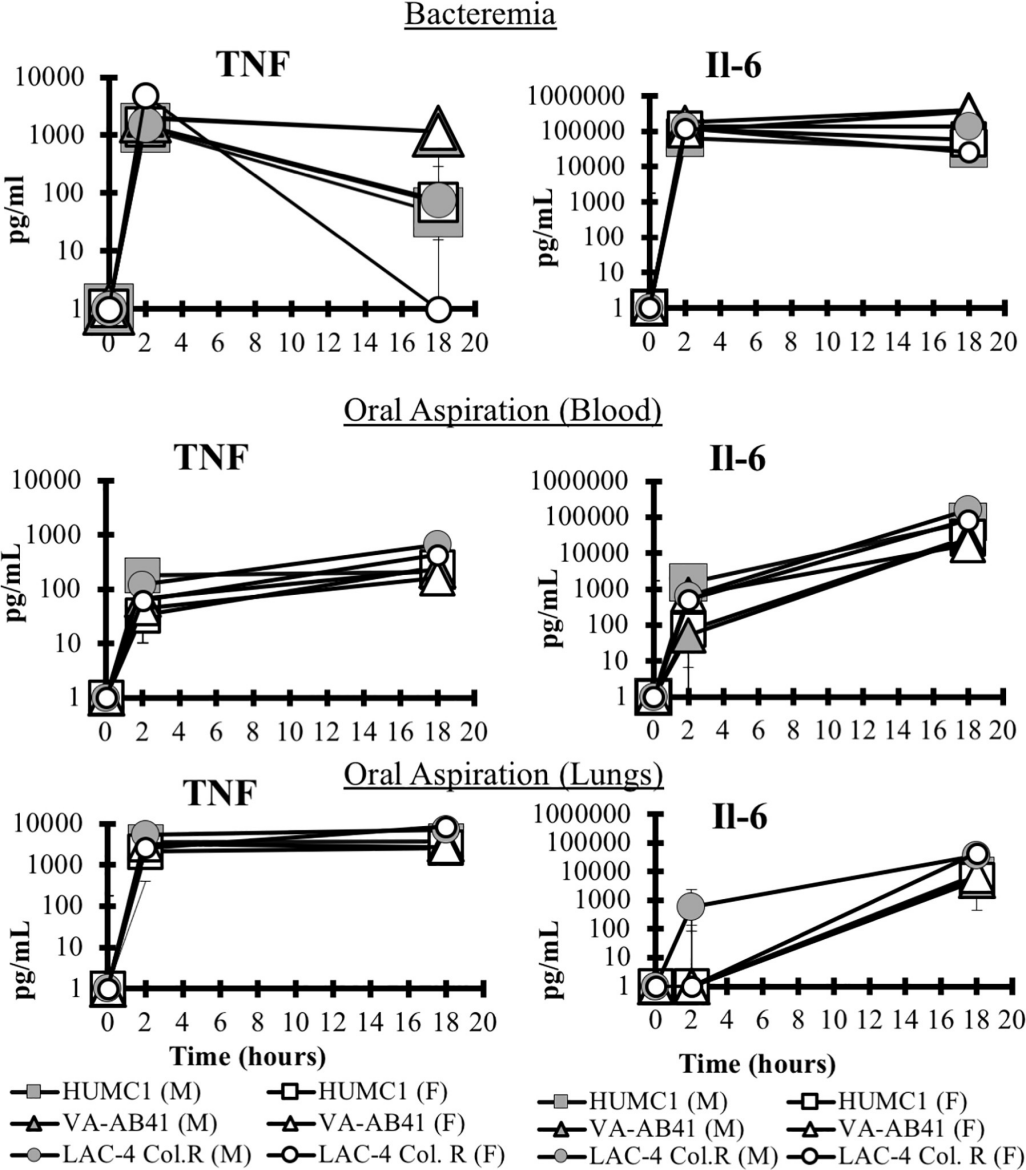
Host immune response. Blood samples were collected from healthy and infected mice at 2 and 18 hours post-infection. TNF was significantly upregulated at 2 hours and 18 hours for all infecting strains in both the bacteremia and OA infection models (P<0.05) but only at 18 hours for IL-6 (P<0.05).

**Fig 7:**
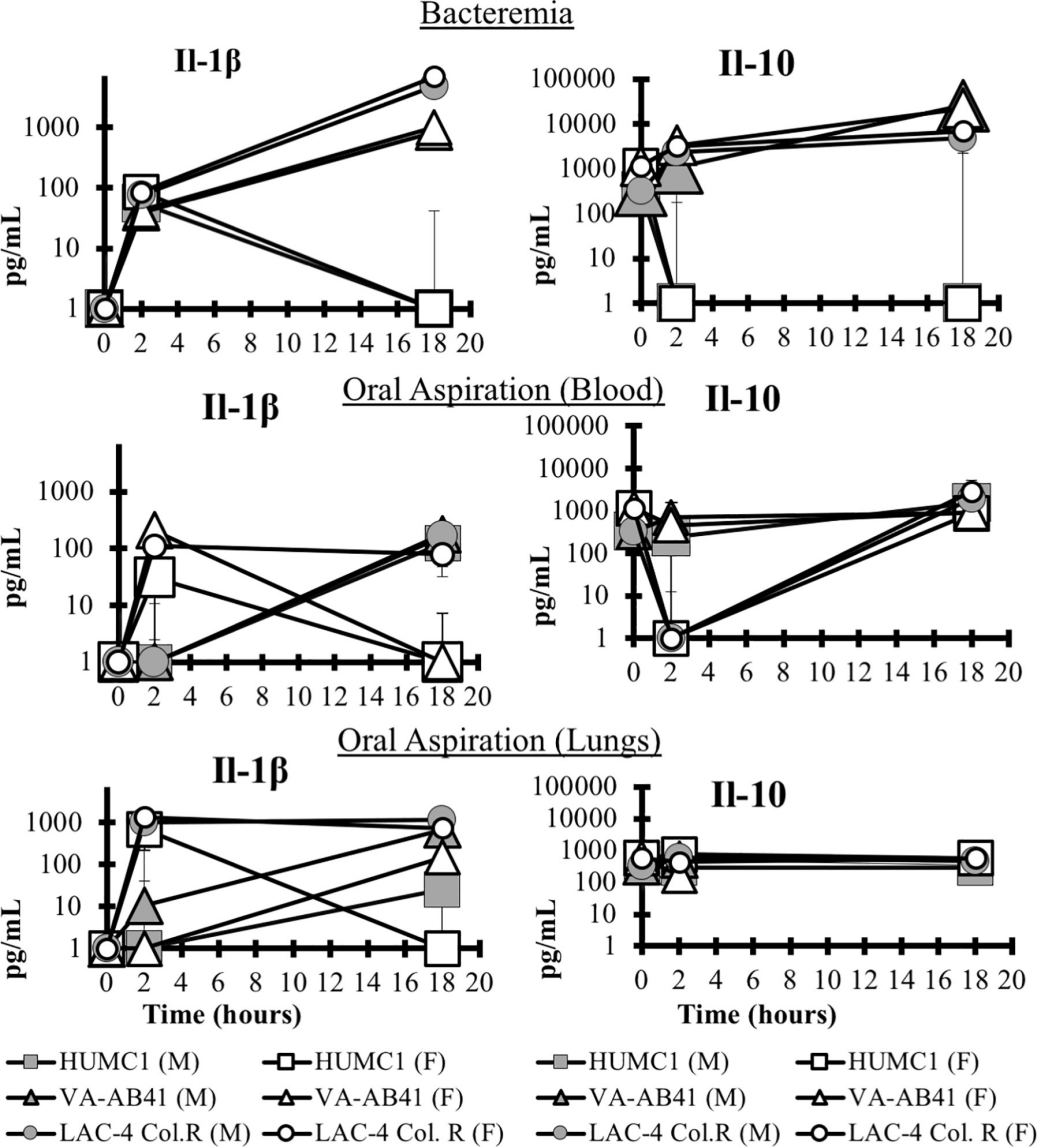
Host immune response. The expression of the pro-inflammatory cytokine Il-1β was more variable and was not significantly upregulated across all conditions. The mice infected with LAC-4 Col^R^ or VA-AB41, the anti-inflammatory cytokine IL-10 was upregulated at 18 hours in the bacteremia model (P<0.05), however there was no significant difference in the OA infection model. This could be due to the lesser disease severity in the OA model as compared to the bacteremia model. Il-6 was significantly upregulated at 18 hours for all infection conditions (P<0.05).

In both models, TNF levels rose dramatically at 2 hours in the blood and lungs (Fig 6). Interestingly such levels began to fall slightly in the bacteremia model by 18 hours, while in the pneumonia model, the levels persisted in both the blood and lung. IL-6 levels rose by 2 hour and persisted at 18 hours in both models in the blood; in the pneumonia model, IL-6 levels in the lung rose in a delayed fashion, so that they significantly elevated by 18 hours, but not at 2 hours in most mice. In contrast, both IL-1β and IL-10 were more variable across the strains and models (Fig 7).

### Oral aspiration pneumonia histopathology

**Fig 8:**
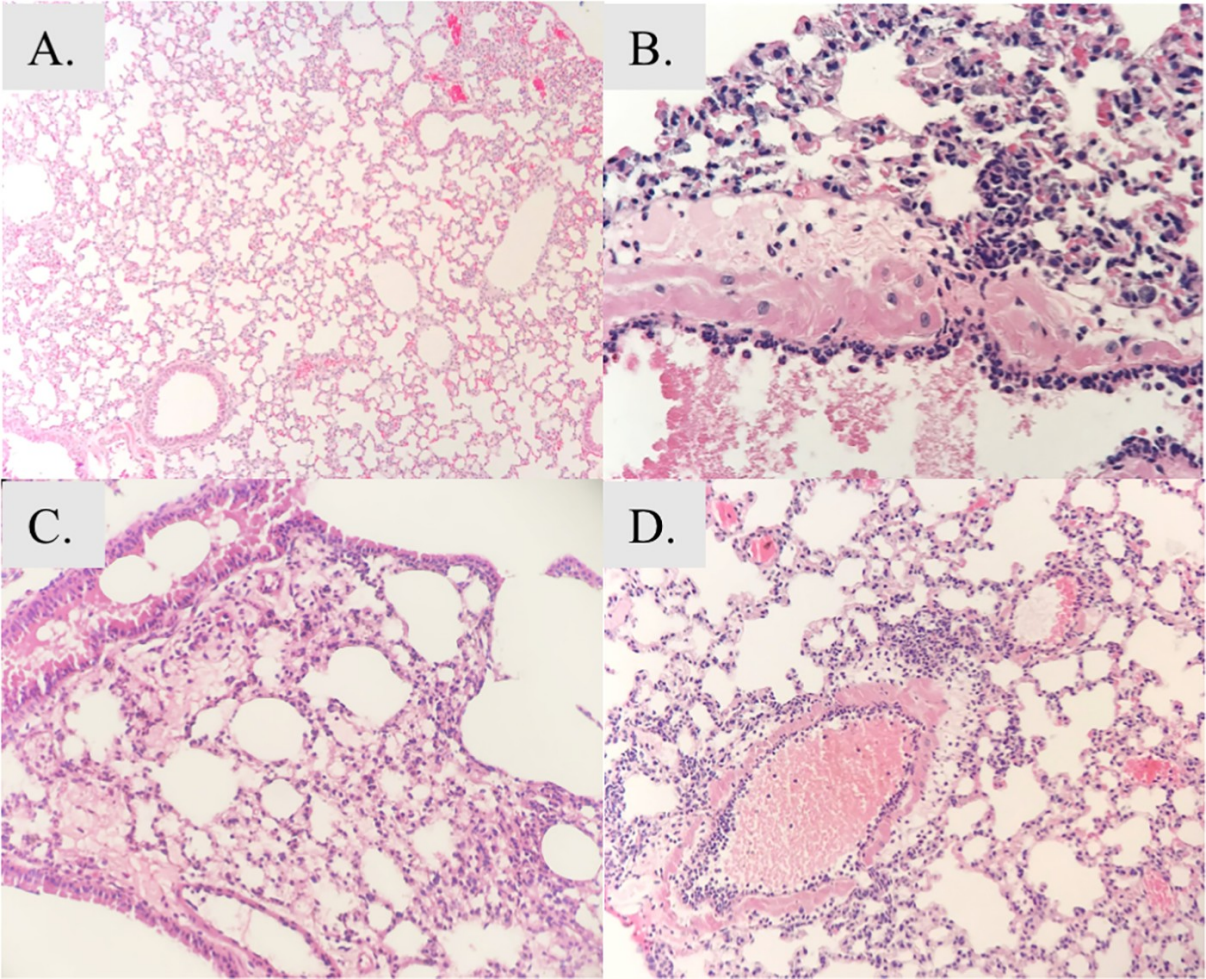
Histopathology of the lungs at 2 hr post infection in the pneumonia model. **A)** Baseline (0 hr) histology of uninfected lung (100X magnification). At 2 hours post-infection mice infected with HUMC1 (**B)**, VA-AB41 **(C)**, and LAC-4 Col^R^ **(D)** demonstrated interstitial edema and neutrophilic inflammation adjacent to the bronchovasculature, with clear alveolae. H&E stain.

**Fig 9:**
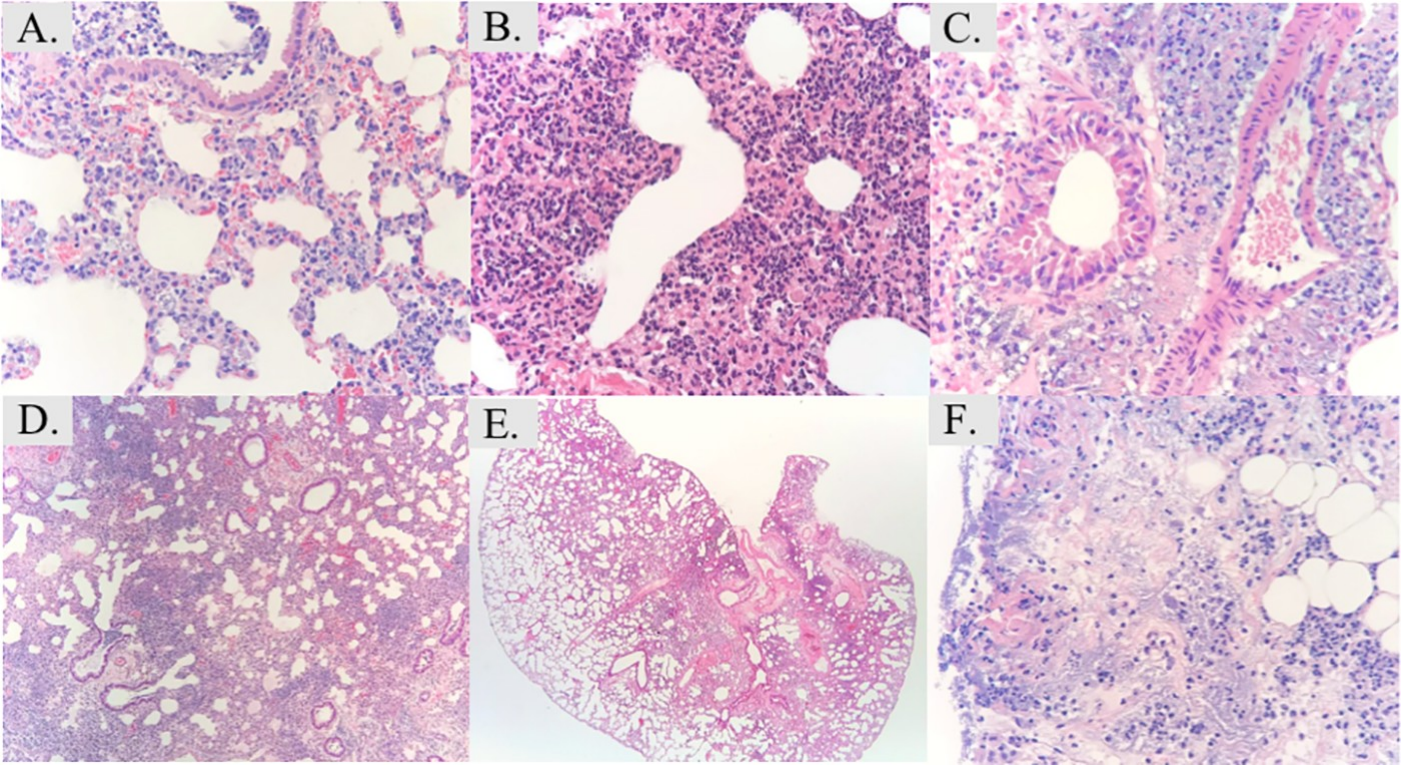
Histopathology of the lungs at 18 hr post infection in the pneumonia model. Severe hemorrhagic pneumonia is seen at 18 hours. **A)** Representative image of HUMC1-infected lung showing relatively mild acute insterstitial inflammation (400X magnification). **B)** VA-AB41 infection resulted in a heavy interstitial neutrophilic response (400X magnification). **C)** In LAC-4 Col^R^ infected lung, heavy organism load is accompanied by an exuberant neutrophilic infiltrate adjacent to bronchovascular structures (400X magnification). **D)** HUMC1-infected lung: low power examination illustrates expansion of interstitium by acute inflammation and distortion of normal air space architecture (50X magnification). **E)** Lung infected by VA-AB41 on low scanning power examination shows central distribution of the infectious process in this mouse model (25X magnification). **F)** LAC-4 Col^R^ lung infection showed areas of tissue necrosis associated with high bacterial burden and acute inflammation (400X magnification).

At 2-hrs infection, mice infected with either strain, and of either gender, had developed interstitial edema, with early neutrophilic extravasation from the blood to the interstitium, but alveoli remained clear (Fig 8). By 18 hours, severe hemorrhagic pneumonia developed in the mice (Fig 9). There were conspicuous bacterial aggregates around the bronchovascular tree associated with tissue destruction/necrosis and abundant neutrophils. This was observed in 8/10 (4/5 males, 4/5 females) of the mice. In the 2 without that pattern, involved tissues showed expansion of interstitium by acute inflammation and small intense aggregates of neutrophils (microabscesses), similar to the response seen in the other 2 strains.

HUMC1 also resulted in heavy acute inflammation, and ranged from a focal, milder process involving the alveolar walls and at times neutrophils in the airways, to a severe and more diffuse process with areas of consolidation (Fig 9D). Although there was centralization of the infection around larger bronchovascular structures, there was less intense disease centrally (generally no bacterial aggregates or necrosis) and further extension of the process peripherally than with LAC-4 Col^R^. One mouse (S1 Table) showed necrosis and bacterial aggregates around bronchi/bronchioles and pulmonary arteries, similar to the LAC-4 Col^R^ group (Fig 9E).

VA-AB41 was more similar to HUMC1 in the distribution of inflammation. There was also a spectrum of severity from focal extension of acute inflammation beyond the central structures to larger areas of exuberant acute inflammation (some with microabscess formation) and involvement of alveolar airspaces. Only one slide showed multifocal areas of necrosis.

## Discussion

We have previously screened over 30 different strains of *A*. *baumannii* and observed considerable variability in their respective virulence in mice.[18] The majority of strains previously tested were not virulent in normal immune-competent C3HeB/FeJ mice and therefore it was important to identify strains with unique and desirable antibiotic-sensitivity profiles that are also virulent in both the bacteremia and oral-aspiration pneumonia disease models.

In this study we describe the clinical, microbiological, and sepsis characteristics of mice lethally infected via the tail-vein or aspiration pneumonia with a panel of strains of *A*. *baumannii* with varying antibiotic susceptibility. These results establish parameters to follow in order to assess efficacy of novel preventative and therapeutic approaches for these infections. Of note, the time to death is significantly more rapid in the bacteremia model. Mice are generally moribund within 24-hours post infection in the bacteremia model but mice are generally moribund at 48 to 72 hours post infection in the oral aspiration pneumonia model. Clinical activity scores were similar between the 2 infection models at the 18-hour time point despite the fact that the mice in the oral aspiration pneumonia model would still be expected to survive an additional 24–48 hours. This underscores the difficulty in identifying sensitive biomarkers that are predictive of outcome.

Bacterial density varied widely by 18 hours post-infection despite the fact that all mice were infected with a lethal inoculum. Thus variances in host response likely drives the fatal outcome as much as bacterial density. However, no specific cytokine was identified that could account for these differences.

The sepsis biomarker results revealed important differences between the bloodstream and pneumonia models of infection, which recapitulate clinical disease. In the bacteremia model, classic septic shock occurs, in which mice develop a metabolic acidosis, characterized by low blood pH, falling bicarbonate, and a rising base deficit. The metabolic acidosis is thus consistent with classical lactic acidosis due to Gram negative bacteremic septic shock. In contrast, during the pneumonia model, the acidosis that occurred was due to a respiratory acidosis, indicated by a low blood pH but with a rising bicarbonate and a falling base deficit. As ventilation failed due to progressive alveolar filling, blood CO2 rose, resulting in increased bicarbonate and hydrogen anions (H_2_O + CO_2_ = HCO_3_^-^ + H^+^). Thus the pneumonia model approximates severe respiratory failure and the bacteremia model severe septic shock.

We observed some gender differences that are informative to pathogenesis and translational research. Female mice are significantly smaller than male mice, and hence using age-matched mice would likely lead to differences in outcomes. We chose to use female mice that were two weeks older than the male mice to enable similar bacterial inocula to be placed in mice with similar body weights. Using weight-matched rather than age-matched mice, we found no differences in outcomes by gender for most strain/model combinations. However, we did find one strain that resulted in differences in survival in the IV model and one other strain with difference in survival in the OA model. Thus validation of each strain in each model is necessary across host genders.

Collectively, these results establish the natural history of clinical, sepsis biomarker, and inflammatory cytokine responses to *A*. *baumannii* bloodstream and lung infection using strain-typed, virulent, clinical isolates with well characterized antimicrobial susceptibility profiles. Assessment of changes induced by novel interventions can be focused on these paramaters. Thus, these results enable future validation of critically needed novel therapeutics against these deadly infections.

## Supporting information

S1 Fig Body weight of uninfected C3H/FeJ mice. No significant difference is observed in the weights of 8-week male mice as compared to 10-week female mice. Median and interquartile ranges are plotted.
(TIFF)

S1 Table Histopathology notes. (XLSX)
