# Supplementary figures and images for: Natural history of Acinetobacter baumannii infection in mice
Source: PLoS One. 2019 Jul 18;14(7):e0219824. doi: 10.1371/journal.pone.0219824 (PMC6638954; doi:10.1371/journal.pone.0219824)

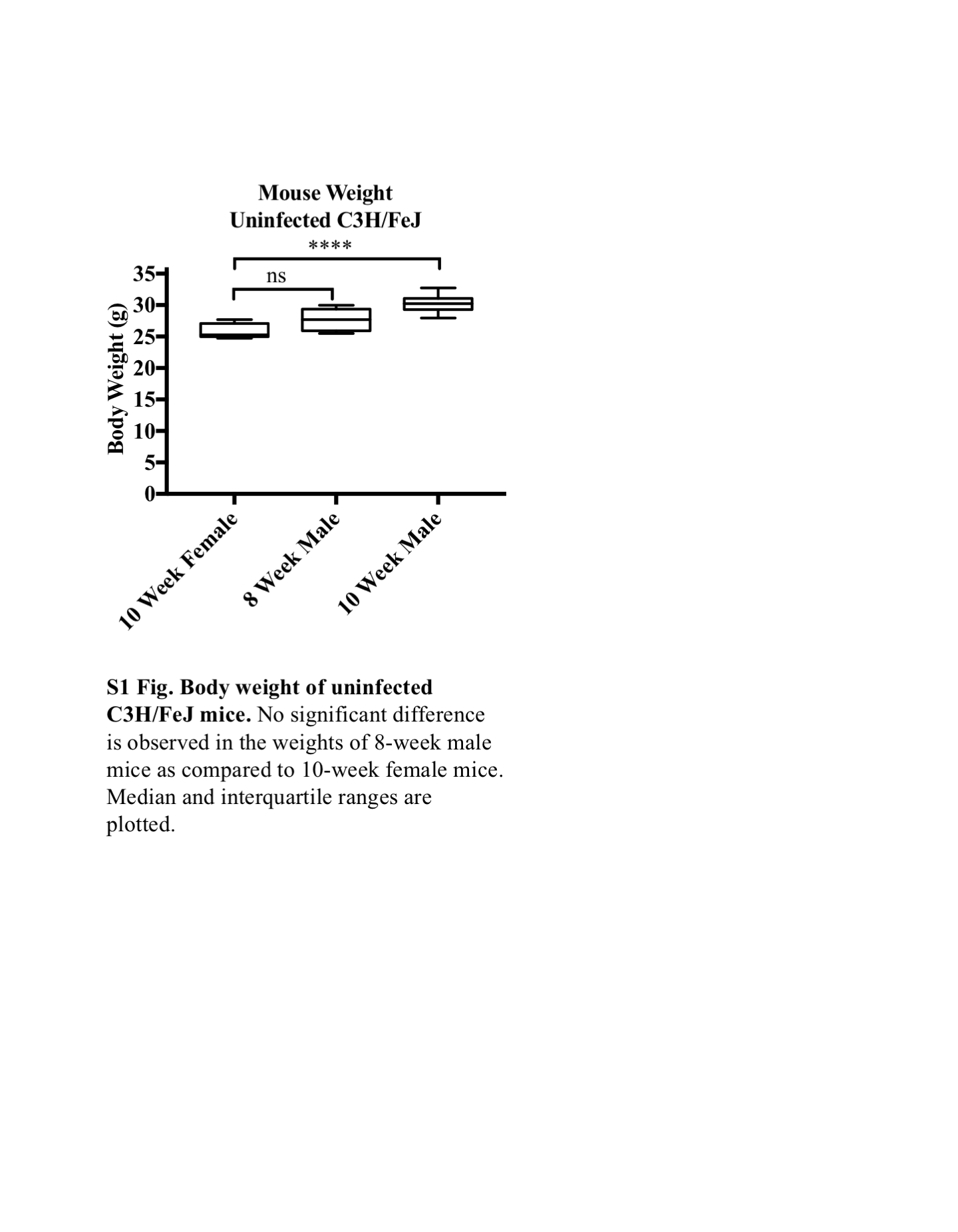

Supplement: S1 Fig — No significant difference is observed in the weights of 8-week male mice as compared to 10-week female mice. Median and interquartile ranges are plotted. (TIFF) [file pone.0219824.s001.tiff]
